# *EDAR*-induced hypohidrotic ectodermal dysplasia: a clinical study on signs and symptoms in individuals with a heterozygous c.1072C > T mutation

**DOI:** 10.1186/1471-2350-15-57

**Published:** 2014-05-16

**Authors:** Catarina Falk Kieri, Birgitta Bergendal, Lisbet K Lind, Marcus Schmitt-Egenolf, Christina Stecksén-Blicks

**Affiliations:** 1 Pediatric Dentistry, Department of Odontology, Faculty of Medicine, Umeå University, Umeå, Sweden; 2 National Oral Disability Centre for Rare Disorders, The Institute for Postgraduate Dental Education, Jönköping, Sweden; 3 Medical and Clinical Genetics, Department of Medical Biosciences, Faculty of Medicine, Umeå University, Umeå, Sweden; 4 Dermatology, Medicine, Department of Public Health and Clinical Medicine, Faculty of Medicine, Umeå University, Umeå, Sweden

**Keywords:** Ectodermal dysplasia, EDAR, Oligodontia, Orofacial function, Saliva, Sweating, Tooth agenesis

## Abstract

**Background:**

Mutations in the *EDAR*-gene cause hypohidrotic ectodermal dysplasia, however, the oral phenotype has been described in a limited number of cases. The aim of the present study was to clinically describe individuals with the c.1072C > T mutation (p. Arg358X) in the *EDAR* gene with respect to dental signs and saliva secretion, symptoms from other ectodermal structures and to assess orofacial function.

**Methods:**

Individuals in three families living in Sweden, where some members had a known c.1072C > T mutation in the *EDAR* gene with an autosomal dominant inheritance (AD), were included in a clinical investigation on oral signs and symptoms and self-reported symptoms from other ectodermal structures (n = 37). Confirmation of the c.1072C > T mutation in the *EDAR* gene were performed by genomic sequencing. Orofacial function was evaluated with NOT-S.

**Results:**

The mutation was identified in 17 of 37 family members. The mean number of missing teeth due to agenesis was 10.3 ± 4.1, (range 4–17) in the mutation group and 0.1 ± 0.3, (range 0–1) in the non-mutation group (p < 0.01). All individuals with the mutation were missing the maxillary lateral incisors and one or more of the mandibular incisors; and 81.3% were missing all four. Stimulated saliva secretion was 0.9 ± 0.5 ml/min in the mutation group vs 1.7 ± 0.6 ml/min in the non-mutation group (p < 0.01). Reduced ability to sweat was reported by 82% in the mutation group and by 20% in the non-mutation group (p < 0.01). The mean NOT-S score was 3.0 ± 1.9 (range 0–6) in the mutation group and 1.5 ± 1.1 (range 0–5) in the non-mutation group (p < 0.01). Lisping was present in 56% of individuals in the mutation group.

**Conclusions:**

Individuals with a c.1072C > T mutation in the *EDAR-*gene displayed a typical pattern of congenitally missing teeth in the frontal area with functional consequences. They therefore have a need for special attention in dental care, both with reference to tooth agenesis and low salivary secretion with an increased risk for caries. Sweating problems were the most frequently reported symptom from other ectodermal structures.

## Background

The term ectodermal dysplasia (ED) has been used to describe around 200 different clinical conditions [1]. EDs are genetic disorders with lack or dysgenesis of at least two of the ectodermal derivatives hair, nails, teeth or sweat glands [2]. Hypohidrotic ectodermal dysplasia (HED) is the most common form of ectodermal dysplasia and is characterized by defective development of teeth, hair, and sweat glands. Common symptoms in subjects with HED are reduced number of teeth and sweat glands, reduced saliva secretion, sparse and thin hair, and dry skin [3]. Other clinical manifestations are dryness of airways and mucous membranes presumably due to the defective development of exocrine glands. HED can also be associated with dysmorphic facial features as a prominent forehead, dark, hyperkeratinized skin around the eyes, everted nose, and prominent lips [4].

HED can be inherited in an X-linked, autosomal dominant or autosomal recessive manner [1]. Four genes (*EDA1, EDAR, EDARADD,* and *WNT10A*) account for 90% of hypohidrotic/anhidrotic ectodermal dysplasia cases [4].

The EDAR protein plays an important role for embryogenesis. The protein is a member of the tumor necrosis factor (TNF) receptor family, and is activated by its ligand EDA and uses EDARADD as an adaptor to build an intracellular NF-kB signal-transducing complex which is necessary for normal development of ectodermal organs both in humans and in mice [5,6], Autosomal dominant (AD) forms of HED have previously been linked to mutations in the ectodysplasin 1 anhidrotic receptor (EDAR) protein but mutations in the *EDAR* gene are also associated with recessive forms of ED [7,8]. Mutations in *EDAR* have been reported to account for 25 per cent of non-EDA1 HED cases [9].

Previously we analyzed the coding sequence of the *EDAR* gene in two families with AD HED and in both families identified a non-sense C to T mutation in exon 12, c.1072C > T, that changes an arginine amino acid into a stop codon; p. Arg358X [10]. Our finding corroborated an earlier finding in an American family [7]. AD HED is not as thoroughly clinically characterized as the more common X-linked HED, although one clinical study in a family with putative AD HED was made in Texas, USA, where affected members of the family had mild hypotrichosis, mild hypodontia and variable degrees of hypohidrosis [11]. Another large American family with known AD HED showed hypotrichosis, hypohidrosis, hypodontia with abnormally shaped teeth, and variable nail abnormalities [12], however, in this study number and type of missing teeth was not reported. Additionally, three successive generations of women with AD HED with a defect of the 2q12 region has been described with alopecia, anhidrosis, hypodontia and malar hypoplasia [13].

The aim of the present study was to clinically describe individuals with the c.1072C > T mutation (p. Arg358X) in the *EDAR* gene with respect to dental signs and saliva secretion, symptoms from other ectodermal structures and to assess orofacial function. A secondary aim was to assess caries experience and dental treatment received and to compare all outcomes with family members without the mutation. The null hypothesis was that there were no differences between family members with and without the c.1072C > T mutation.

## Methods

### Subjects

**Figure 1:**
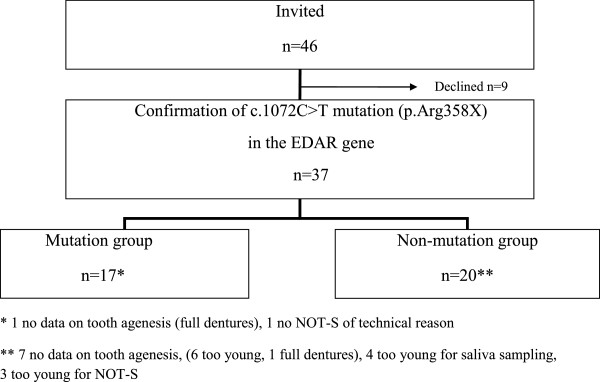
Flow chart and study design.

**Figure 2:**
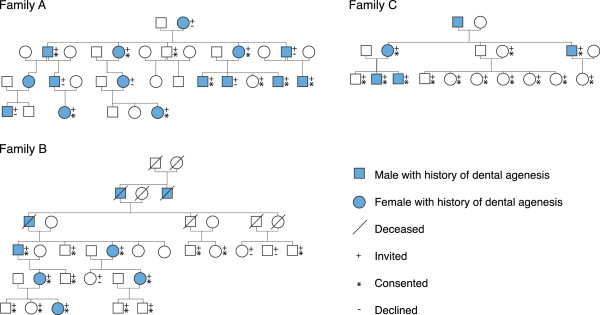
Pedigrees of included families.

Individuals in two families living in northern Sweden, where some members had a known mutation in the *EDAR* gene, were contacted and invited to participate in a clinical examination for patient characterization (n = 33). Fourteen of the family members had earlier been included in a genetic study [10] where a premature stop codon in the *EDAR* gene was found. These two families were from the same geographical region in northern Sweden. Additionally, 13 members of a third family who lived in the middle part of Sweden, where the same mutation had been identified in two individuals, were contacted and invited to participate. Nine members in the two northern families declined to participate. In the third family all invited members were examined. A total of 37 individuals participated (Figure 1). Pedigrees of the included families are shown in Figure 2.

Written informed consent was received from all participating individuals. Parents provided consent for individuals below 18 years of age. The study was approved by The Regional Ethical Review Board in Umeå, Sweden (Dnr 2011-123-32 M).

### Confirmation of the c.1072C > T mutation in the EDAR gene

Buccal swabs were collected from all participating individuals and DNA was prepared according to standard procedures. PCR of EDAR exon 12 was accomplished with flanking primers F’-GACCTTCTATTGACTGTGACTTGC and R’-CAGTCTTTTGGCACCACTCA [10]. PCR reactions were run in a PTC-100 thermo cycler (MJ Research Inc, Watertown, MA) using KAPA2G Robust HotStart PCR Ready Mix (2X) (Kapa Biosystems, Boston, MA) and presence of PCR products was verified by agarose electrophoresis. Sequencing of the PCR products was performed at MWG Eurofins, Germany.

### Oral examination and questionnaire

In an oral clinical examination the number and type of teeth missing from agenesis were recorded, with access to existing dental x-rays from the dental clinics where the subjects were registered. Two extra-oral and five intra-oral-photographs were taken of each participant. In connection with the oral examination the participants or parents of young children were asked to fill out a questionnaire on any inconvenience from other ectodermal structures apart from teeth. Six subjects were too young for confirmation of tooth agenesis, however none of these children had the c.1072C > T mutation. In two subjects who were edentulous and were wearing dentures, of which one had the mutation, it was not possible to obtain information on tooth agenesis. Decayed including cavitated caries lesions, missing and filled surfaces (dmfs and DMFT) were recorded. For molars and premolars extracted due to caries 4 surfaces, and for canines and incisors three surfaces, were counted in the dmfs/DMFS index. Correspondingly, for teeth with crowns the same number of surfaces was included in the dmfs/DMFS index. The number of abutment teeth in fixed dental prostheses and the number of dental implants were also recorded.

### Saliva

Chewing stimulated whole saliva was collected during five minutes and the volume was read and registered in ml/min. Saliva samples were obtained from 33 subjects. In four subjects no saliva samples could be obtained; three were children aged zero to 4 years, and one was a man 73 years of age with complete dentures. None of these subjects had the c.1072C > T mutation in the *EDAR* gene.

### Screening of orofacial function

The Nordic Orofacial Test-Screening (NOT-S) was used to assess orofacial function. This method is applicable from three years of age and has been validated and shown to discriminate well between healthy individuals and persons with chronic diseases and disabilities as well as ectodermal dysplasias [14,15]. The protocol contains twelve domains of orofacial function – six assessed through interview and six through clinical observation as the participant performs various tasks using a picture manual. The six interview domains are: Sensory function, Breathing, Habits, Chewing and swallowing, Drooling, and Dryness of the mouth. The six domains assessed in the clinical examination are: Face at rest, Nose breathing, Facial expression, Masticatory muscle and jaw function, Oral motor function, and Speech. A positive answer in a domain gives 1 point, the maximum score being 12 points. Two additional symptoms, lisping and hoarseness were also evaluated. In three subjects in the non-mutation group screening of orofacial function could not be performed due to young age, and in one 13-year-old girl in the mutation group because of technical reasons.

#### ***Statistical analysis***

Data were subjected to statistical analysis using PASW statistics software (version 20.0, Chicago, IL, USA SPSS. Categorical data were analysed with chi-square test, and continuous data with ANOVA. A p-value of less than 0.05 was considered as statistically significant.

## Results

The c.1072C > T mutation was identified in 17 of the 37 included members from the three families. The mean age of participants was 31.5 ± 23.1 years (range 0–78 years); 35.6 ± 22.6 in the mutation group and 28.2 ± 23.7 in the non-mutation group (p > 0.05); 49% were males, 47% in the mutation group and 50% in the non-mutation group (p > 0.05).

### Missing teeth from tooth agenesis and aberrant tooth form

**Figure 3:**
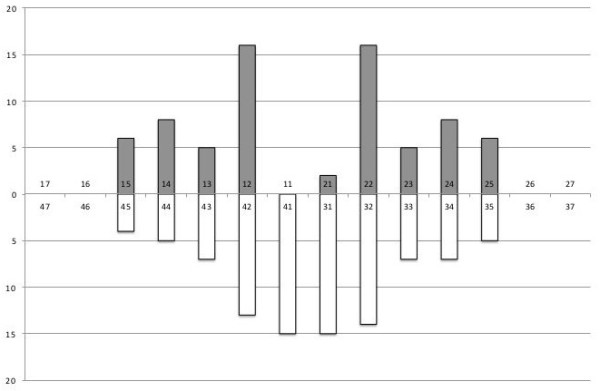
**Missing teeth in 16 subjects with a c.1072C > T mutation (p. Arg358X) in the *EDAR *gene.** Y-axis denotes number of subjects. Tooth numbers are indicated along the X-axis.

**Figure 4:**
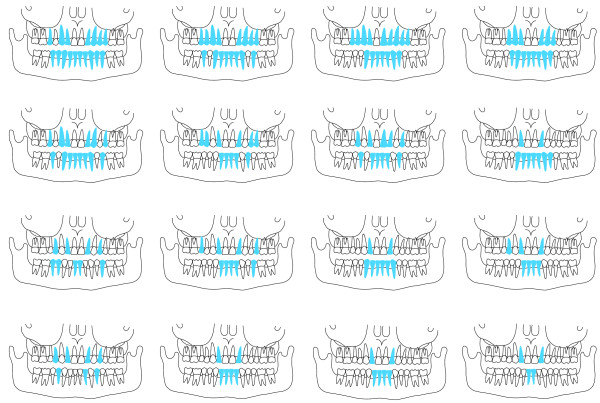
**Dentograms of teeth missing from agenesis in order of frequency in 16 subjects with a c.1072C > T mutation (p. Arg358X) in the EDAR gene.** Missing teeth are indicated in blue colour.

The mean number of missing teeth from agenesis was 10.3 ± 4.1, (range 4–17) in the mutation group and 0.1 ± 0.3, (range 0–1) in the non-mutation group (p < 0.01), (Table 1). Number and type of missing teeth is shown in Figure 3 and displayed in individual dentograms in Figure 4. The most commonly missing teeth were the upper lateral incisors which were missing in all individuals in the mutation group, followed by the lower incisors, out of which all were missing at least one, and 13 (81.3%) were missing all four. All but one individual in the mutation group had oligodontia defined as the congenital missing of six or more teeth, wisdom teeth excluded. In the mutation group males were missing 9.8 ± 4.2 teeth and females 10.7 ± 4.2 teeth (p > 0.05). No difference in the pattern of missing teeth was seen between males and females. Teeth with an aberrant form were seen in 64% in the mutation group compared to 6% in the non-mutation group (p < 0.01).

**Table 1:** Signs from teeth, saliva secretion, dmfs/DMFS, abutment teeth and dental implants in the mutation and non-mutation group

	**Mutation group**	**Non-mutation group**	**p**
**Missing permanent teeth**	10.3, (4.1), 8.1-12.3, 4-17	0.1, (0.3), -0.1-0.2, 0-1	<0.01
mean (sd), 95% CI, min-max			
**Aberrant tooth form **%	64	6	<0.01
**Saliva secretion**	0.9 (0.5), 0.7-1.2, 0.4-2.0	1.7 (0.6), 1.4–2.0, 0.8-2.6	<0.01
ml/min mean (sd), 95% CI, min-max			
**dmfs**	4.5, (7.1), 0.1-8.8, 0-25	0	<0.05
mean (sd), 95% CI, min-max			
**DMFS**	18.1, (18.1), 8.0-28.1, 0-61	20.0, (24.1), 7.2-32.8, 0-71	n.s
mean (sd), 95% CI, min-max			
**Abutment teeth**	3.1, (2.9), 1.4-4.7, 0-8	0.2, (0.6), -0.2-0.5, 0-2	<0.01
mean (sd), 95% CI, min-max			
**Abutment primary teeth**	1.1, (1.4), 0.2-2.0, 0-4	0	<0.05
mean (sd), 95% CI, min-max			
**Number of implants**	1.0, (1.6), 0.1-1.9, 0-5	0	<0.05
mean (sd), 95% CI, min-max			

### Saliva secretion

There was a statistically significant difference in stimulated saliva secretion, 0.9 ± 0.5 ml/min in the mutation group vs 1.7 ± 0.6 ml/min in the non-mutation group (p < 0.01), (Table 1). The correlation between saliva secretion and self-reported dry mouth was statistically significant (r = 0.5, p < 0.05).

### Signs and symptoms from other ectodermal structures

Reduced ability to sweat was reported by 82% in the mutation group and by 20% in the non-mutation group (p < 0.01), (Table 2). No statistically significant differences were reported for asthma and allergies, air-way infections, symptom from skin and nails (p > 0.05).

**Table 2:** Reported signs and symptoms from other ectodermal structures in the mutation and non-mutation group

	**Mutation group**	**Non-mutation group**	**p**
	**n = 17**	**n = 20**	
	**%**	**%**	
Allergy, asthma	24	10	n.s
Air-way infections	29	15	n.s
Skin problems	59	40	n.s
Nail problems	29	5	n.s
Sweating difficulties	82	20	<0.01

### Orofacial function

**Figure 5:**
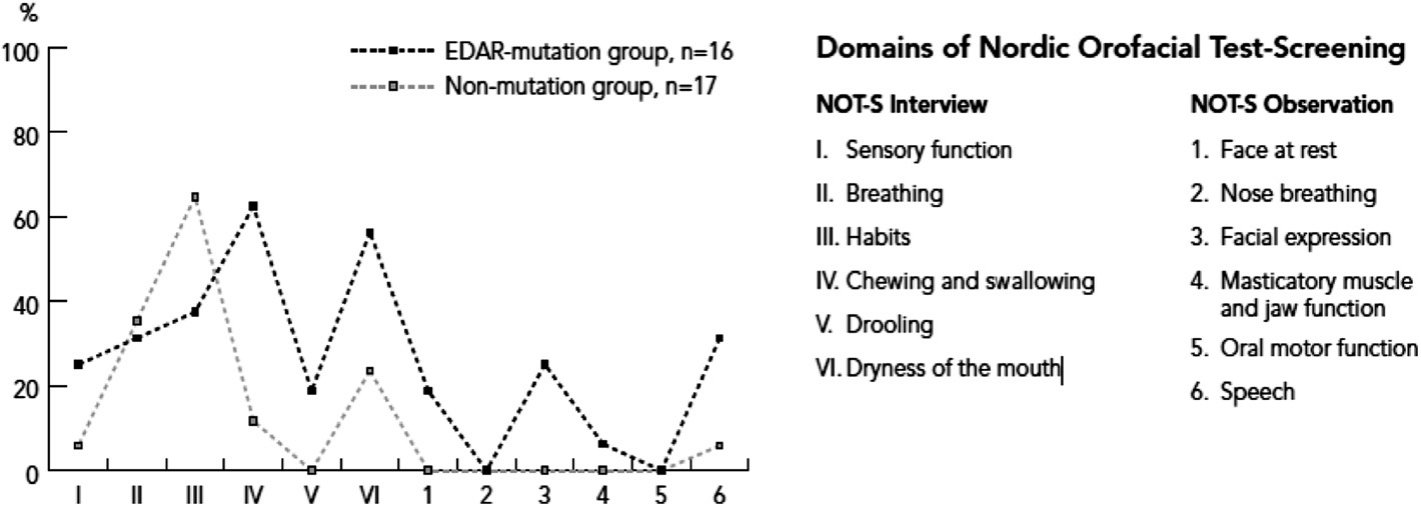
Orofacial dysfunction profiles based on frequencies of NOT-S domain scores (%) in the EDAR-mutation group (n = 16) and the non-mutation group (n = 17), respectively.

The mean NOT-S score was 3.0 ± 1.9 (range 0–6) in the mutation group and 1.5 ± 1.1 (range 0–5) in the non-mutation group (p < 0.01). The results for the two groups are illustrated as dysfunction profiles in Figure 5. In the mutation group the most frequent domains with scores were in order of frequency Chewing and swallowing (62.5%), Dryness of the mouth (56.3%), Habits (37.5%), and Breathing and Speech (31.3%). In the non-mutation group two domains where more than 30% of the subjects had scores were Habits (64.7%) and Breathing (35.3%). The two additional symptoms that were evaluated, hoarseness and lisping, were registered in three (19%) and nine (56%) individuals in the mutation group, respectively, and in no individual in the non-mutation group.

### dmfs/DMFS, number of abutment teeth and dental implants

The mean dmfs was 4.5 ± 7.1 (range 0–25) in the mutation group and 0 in the non-mutation group (p < 0.05), (Table 1). No statistically significant differences were reported for DMFS (p > 0.05). Excluding two children, 6 and 8 years of age, oral habilitation had been performed in 12 individuals in the mutation group (80%). Of these, nine had tooth-supported fixed dental prostheses and five of them had also primary teeth as abutment teeth. Six subjects had implant-supported fixed dental prostheses, or combined tooth- and implant supported restorations. In the non-mutation group there were two individuals with one abutment tooth each. Statistically significant differences were displayed in number of abutment teeth (p < 0.01), and number of dental implants (p < 0.05), (Table 1).

## Discussion

Overall, the signs and symptoms from ectodermal structures associated with c.1072C > T heterozygous mutation in the EDAR gene were generally milder than what have been linked with the more common EDA1 mutations [4]. EDAR is part of a signaling cascade leading to the development of teeth and other ectodermal structures [9]. In HEDs there is an improper interaction between ectoderm and mesenchyme which results in dysplastic abnormalities in tissues derived from the ectoderm. Mutations in different genes typically cause phenotypes characteristic in terms of severity and affected teeth [16]. The effect of an impaired EDAR protein function is clearly shown in the three families studied. An advantage of this study is that the genetic background in the members of each family is as similar as possible. The signs and symptoms presented in the mutation group are thus almost certainly due to the c.1072C > T mutation, and as family members with and without the p. Arg358X alteration could be seen to differ in number of missing teeth, tooth form, saliva secretion and sweating ability the null hypothesis of no difference is firmly rejected. Of the individuals participating in this study 46% had inherited the mutated EDAR allele from their affected parent which agrees well with the expected risk of 50% inheritance in an AD disorder.

Clinical and radiographic confirmation of tooth agenesis was achieved in all subjects carrying the mutation except in a 73 year-old man with dentures where reliable clinical data were not available. Although there was considerable variation in number of missing teeth, all persons with the c.1072C > T mutation in the *EDAR*-gene were missing both maxillary lateral incisors and at least one mandibular incisor. Seven individuals (43.8%) had a continuous edentulous space in the mandibular frontal area varying from six to 10 teeth. The most stable teeth were molars in both jaws, out of which none was missing. The second most stable teeth were the maxillary central incisors, out of which two individuals were missing one each.

Saliva secretion was lower in the mutation group and 47% in this group had a stimulated saliva secretion of 0.7 ml/min or less compared to 0% in the non-mutation group. Previously, a negative correlation between number of teeth missing from agenesis and saliva secretion was shown in subjects with oligodontia [17] but this observation was not confirmed in the present study. Although there was no difference in caries experience in permanent teeth between the mutation and non-mutation group, salivary secretion tests is recommended in persons with known or suspected EDs [18]. The low saliva secretion in the mutation group increases the caries risk unless other etiological factors are restricted or under control [19].

Self-reported symptoms from other ectodermal structures varied but were generally mild, sweating problems being the most frequently reported. The clinical expression of the c.1072C > T mutation in affected individuals in the three families might vary due to random combinations of normal and truncated EDAR protein. This can lower the number of functional trimeric complexes that are necessary for proper interactions with EDARADD. Thus the proportion of functional EDAR homo-trimers in different individuals may vary both during embryogenesis and later developmental stages and consequently a variation in the level of proper cell signalling in the ectoderm can result. Alternatively, the dominant negative effect of the c.1072C > T mutation might result from an altered subcellular location of the truncated protein as compared to the native EDAR protein, in analogy with observations of the orthologous mutated and wildtype Edar protein in mouse cell cultures [20]. A third scenario might be changes in the interactions between the truncated Arg358Ter EDAR protein, as compared to full length EDAR, and different variants of the ectodysplasin and EDARADD proteins.

The orofacial function as evaluated with the NOT-S protocol showed higher mean total NOT-S scores in the mutation group, 3.0, vs 1.5 in the non-mutation group. The most common domains with scores in the mutation group were Chewing and swallowing (62.5%) and Dryness of the mouth (56.3%), whereas almost one third (31.3%) had scores in the Speech domain. This is in accordance with a study in individuals with different forms of ED, where 32 individuals with hypohidrotic ED had similar outcomes with a mean total NOT-S score of 3.0 (range 0–6) [15]. The domains with most frequent scores in that study were Chewing and swallowing (81.3%), Dryness of the mouth (43.8%) and Speech (34.4%). Since all but three individuals in the mutation group missed all mandibular incisors, the results indicate that the development of a clear speech seems to be more difficult and this may thus be a secondary effect of defective tooth development during embryogenesis. Lisping was found only in the mutation group with more than half of group with the problem. In the aforementioned study of NOT-S in ED [15], among individuals with HED 43.8% had a lisp. In many cases speech is improved after provision of artificial teeth in the lower frontal area [21,22]. Early recognition of tooth agenesis is important in order to accomplish interceptive measures and to carry out a plan for orthodontic treatment and later tooth replacement [23]. For individuals with many missing teeth there is a high need of oral habilitation.

## Conclusion

Individuals with *EDAR*-induced AD HED displayed a typical pattern of missing teeth in the frontal area, with functional consequences. They therefore have a need for special attention in dental care, both with reference to tooth agenesis, low salivary secretion with an increased risk for caries, and a need for oral habilitation. Among symptoms from other ectodermal structures sweating problems were the most frequently reported.

## Competing interests

The authors declare that they have no competing interests.

## Authors’ contributions

CFK performed the clinical registrations, computerized data and drafted the manuscript together with CSB. BB contributed with illustrations and revised the manuscript critically. LL analysed the genetic results and revised the manuscript critically. MSE designed the study, contributed with illustrations and revised the manuscript critically. CSB designed the study, performed the statistical data analysis, drafted the manuscript together with CFK and revised it critically. All authors read and approved the final manuscript.

## Pre-publication history

The pre-publication history for this paper can be accessed here:

http://www.biomedcentral.com/1471-2350/15/57/prepub
